# Changes in brain connectivity related to the treatment of depression measured through fMRI: a systematic review

**DOI:** 10.3389/fnhum.2015.00582

**Published:** 2015-11-03

**Authors:** Esteve Gudayol-Ferré, Maribel Peró-Cebollero, Andrés A. González-Garrido, Joan Guàrdia-Olmos

**Affiliations:** ^1^Facultad de Psicología, Universidad Michoacana de San Nicolás de HidalgoMorelia, Mexico; ^2^Departament de Metodologia de les Ciències del Comportament, Facultat de Psicologia, Institut de Recerca en Cervell, Cognició i Conducta IR3C, Universitat de BarcelonaBarcelona, Spain; ^3^Instituto de Neurociencias, Universidad de GuadalajaraGuadalajara, Mexico

**Keywords:** depression, depression treatment, brain connectivity, fMRI, antidepressants

## Abstract

Depression is a mental illness that presents alterations in brain connectivity in the Default Mode Network (DMN), the Affective Network (AN) and other cortical-limbic networks, and the Cognitive Control Network (CCN), among others. In recent years the interest in the possible effect of the different antidepressant treatments on functional connectivity has increased substantially. The goal of this paper is to conduct a systematic review of the studies on the relationship between the treatment of depression and brain connectivity. Nineteen studies were found in a systematic review on this topic. In all of them, there was improvement of the clinical symptoms after antidepressant treatment. In 18 out of the 19 studies, clinical improvement was associated to changes in brain connectivity. It seems that both DMN and the connectivity between cortical and limbic structures consistently changes after antidepressant treatment. However, the current evidence does not allow us to assure that the treatment of depression leads to changes in the CCN. In this regard, some papers report a positive correlation between changes in brain connectivity and improvement of depressive symptomatology, particularly when they measure cortical-limbic connectivity, whereas the changes in DMN do not significantly correlate with clinical improvement. Finally, some papers suggest that changes in connectivity after antidepressant treatment might be partly related to the mechanisms of action of the treatment administered. This effect has been observed in two studies with stimulation treatment (one with rTMS and one with ECT), and in two papers that administered three different pharmacological treatments. Our review allows us to make a series of recommendations that might guide future researchers exploring the effect of anti-depression treatments on brain connectivity.

## Introduction

Major Depressive Disorder (MDD) and other depressive mood disorders present numerous structural and functional alterations of the encephalon associated both to the physiopathology of depression and its diverse symptomatic manifestations (Rogers et al., 2004; Fitzgerald et al., 2014; Wise et al., 2014). Thus, depressive syndromes have been consistently related to reductions in brain volume in several areas related to the regulation of mood such as the anterior cingulate cortex (ACC), along with certain regions from the medial prefrontal cortex (mPFC), e.g., the orbitofrontal cortex (OFC) and the ventromedial cortex (vmPFC). Reductions have also been found in the volume of the lateral prefrontal cortex (LPFC), the basal ganglia, and the hippocampus, among other structures (Wise et al., 2014).

Likewise, functional Magnetic Resonance Imaging (fMRI) allowed us to spot differences in the activation of these and other brain areas, so that, in depressive symptoms, we find—rather consistently—an increase in the activation of the mPFC, the amygdala, and the hippocampus in depressed subjects with respect to the control subjects (Rose et al., 2006; Siegle et al., 2007; Wise et al., 2014). It is likewise common for depressed patients to show fewer activations than healthy persons on the LPFC, the inferior parietal lobe (BA 40), the posterior cingulate cortex (PCC), and the striatum, among other structures (Rose et al., 2006; Siegle et al., 2007; Wise et al., 2014).

Most fMRIs on depression compare the activation found in the cerebral regions above mentioned, among others, to that observed in healthy persons (Chen et al., 2008). However, in recent years, the studies on functional and effective connectivity have revealed that the several brain structures related to behavior do not work in isolation, but they form complex functional integration networks (Friston, 2011). Thus, the brain structures altered in depression are part, in turn, of several connectivity networks. Although the different papers on connectivity in depression sometimes show contradictory results, most of the evidence in the literature suggests that, in MDD, connectivity networks at rest and the connectivity networks activated during specific tasks are all altered (Wang et al., 2012). Accordingly, affective disorders have been linked to alterations of the Default Mode Network (DMN), the Affective Network (AN), the Salience Network (SN), and the Cognitive Control Network (CCN), among others (Dutta et al., 2014).

DMN is a network that becomes active when we are not conducting complex cognitive tasks. DMN includes frontal areas such as the ventromedial prefrontal cortex (vmPFC) and portions of the anterior cingulate cortex (ACC), and from the orbitofrontal cortex (OFC) and parietal areas like the posterior cingulate cortex (PCC) and the medial, lateral, and inferior parietal cortex (Raichle et al., 2001; Greicius et al., 2003). It has been linked to self-reference processes and their alteration; while the pathological interactions of DMN with other networks such as the SN and the CCN would be linked to the states of pathological rumination frequently presented by depressed patients (Broyd et al., 2009; Belleau et al., 2014; Jacobs et al., 2014). It is undoubtedly the most widely studied network in mood disorders and the studies generally show that DMN is hyperactivated in depressed patients (Greicius et al., 2007; Liston et al., 2014). Nevertheless, some papers report the opposite pattern in MDD (Veer et al., 2010; see Wang et al., 2012 for a review), and some studies report hyperactivation and hypoactivation patterns at the same time between different structures within DMN (Wu et al., 2011).

The AN is formed by connections between the ACC, the amygdala, the hypothalamus, and other limbic structures. It has also been reported as altered in MDD (Sheline et al., 2010; Salomons et al., 2014), and since its alterations are involved in hunger, sleep, and sexual conduct, it has been related to the presence of vegetative symptoms in depression (Sheline et al., 2010). It has also been found that depression could present patterns of hypoconnectivity between cortical-limbic structures when the brain is actively processing affective information (Anand et al., 2005; Chen et al., 2008).

The CCN is formed by frontal areas, specifically the dorsolateral prefrontal cortex (DLPFC) and the dorsal anterior cingulate cortex (dACC), and parietal posterior areas, mostly the posterior cingulate cortex (PCC). It is involved in the top-down or goal-directed regulation of attention and in the regulation of working-memory (Corbetta and Shulman, 2002) and it also includes medial temporal lobe parts (Greicius et al., 2009). This network becomes impaired in depression (Rogers et al., 2004; Fales et al., 2008). Some studies show that depressed patients present less connectivity in this network than control subjects (Aizenstein et al., 2009; Liston et al., 2014).

Further evidence suggests that, in depressive disorders, there are alterations of functional connectivity at rest between structures that are part of different networks, as well as in coupling them. For example, the alteration of connectivity between cortical-limbic structures such as the connection between the amygdala and ACC, and the amygdala and DLPFC, among others, has been related to the cognitive biases and neuropsychological alterations of depression (Thomas and Elliott, 2009). It has likewise been suggested that the hyperconnectivity of DMN and the SN with CCN is related to rumination in depression (Jacobs et al., 2014). In addition to the studies of the networks at rest in patients with MDD, further anomalies have been described in brain connectivity, such as alterations in cerebellum-brain connectivity in depressed patients (Guo et al., 2013), or alterations in the inter-hemispheric functional coordination related to disturbances in the connectivity between both cerebral hemispheres (Wei et al., 2014). Likewise, alterations of the connectivity in the affective network (AN) have been reported in depressed patients, as well as between cortical-limbic structures in emotional processing paradigms in depression (Anand et al., 2005; Chen et al., 2008; Delaveau et al., 2011). In addition, a recent meta-analysis by Kaiser et al. (2015) included 27 seed-based voxel-wise connectivity studies in MDD. That study confirmed the alterations in the connectivity of both rest networks and in network coupling. More specifically, it concluded that this illness is characterized by several connectivity alterations in both networks and in network coupling. More specifically, patients with MDD presented hypoconnectivity within the frontoparietal network, hypoconnectivity between frontoparietal systems and parietal areas of the dorsal attention network, hyperconnectivity within the DMN, and hyperconnectivity between frontoparietal control areas and regions of the default network (Kaiser et al., 2015). The authors also provided a model in which the widespread network dysfunction underlies core affective and cognitive alterations in MDD (Kaiser et al., 2015).

Given the generalized alterations of brain connectivity in depression, in recent years there has been substantially increasing interest in the possible effect of the different antidepressive treatments on functional connectivity in this illness, although this phenomenon has been much less studied. Studies on functional neuroimaging show that antidepressive treatment is capable of normalizing brain activations in depressed patients during affective tasks in areas such as DLPFC, Dorsomedial Prefrontal Cortex (DMPFC), and ventrolateral Prefrontal Cortex (VLPFC), among others (Delaveau et al., 2011). Moreover, a recent meta-analysis by Ma (2014) studied the effects of antidepressants on brain activity underlying emotional processing. Their results showed that antidepressant treatments had effects on the activation of limbic core structures such as the amygdala, the thalamus, and ACC, and in other emotional processing structures like MPFC, the insula, and the putamen. Antidepressant medication increased the activity of these structures when the subjects processed positive emotions, whereas the same medication decreased the activity of the same structures while processing negative emotions. For these reasons, it is perfectly plausible to expect that, after the same treatments, the depressed patients will present changes in connectivity related to illness improvement. Accordingly, there is increasing interest in knowing the effect of antidepressants and other somatic treatments for depression on brain connectivity.

The goal of this review is to conduct a systematic review of the studies focusing on the relationship between the treatment of depression and brain connectivity to try to identify some common patterns. Accordingly, we will review the changes in brain connectivity that occur in DMN and CCN after antidepressant treatment, and the changes among other cortical-limbic connections in studies at rest and in studies of activation during emotional tasks. Likewise, we will review some papers measuring changes in connectivity in other brain structures. Additionally, we will review, where possible, the relationship between changes in brain connectivity and clinical improvement observed after antidepressive treatment. To conclude, and also where possible, we will review the possible “causal” relationships between the type of treatment administered and the specific connectivity changes observed when using a particular treatment in MDD.

## Materials and methods

### Article selection

The articles were selected after a search in the following databases: Pubmed, Psychinfo, and Google Scholar. To locate the papers, the following keywords were searched: “depression treatment,” “connectivity,” and “fMRI” or “functional Magnetic Resonance Imaging.” The term “depression treatment” was replaced by “unipolar depression treatment” and “major depressive disorder treatment” and the previous terms were combined with the Boolean link “and” in several bibliographical searches. Such process was conducted by two independent researchers who reproduced the paper selection process. The initial level of agreement was of 98% and they concurred the decision to include or not they articles on which they disagreed.

**Figure 1:**
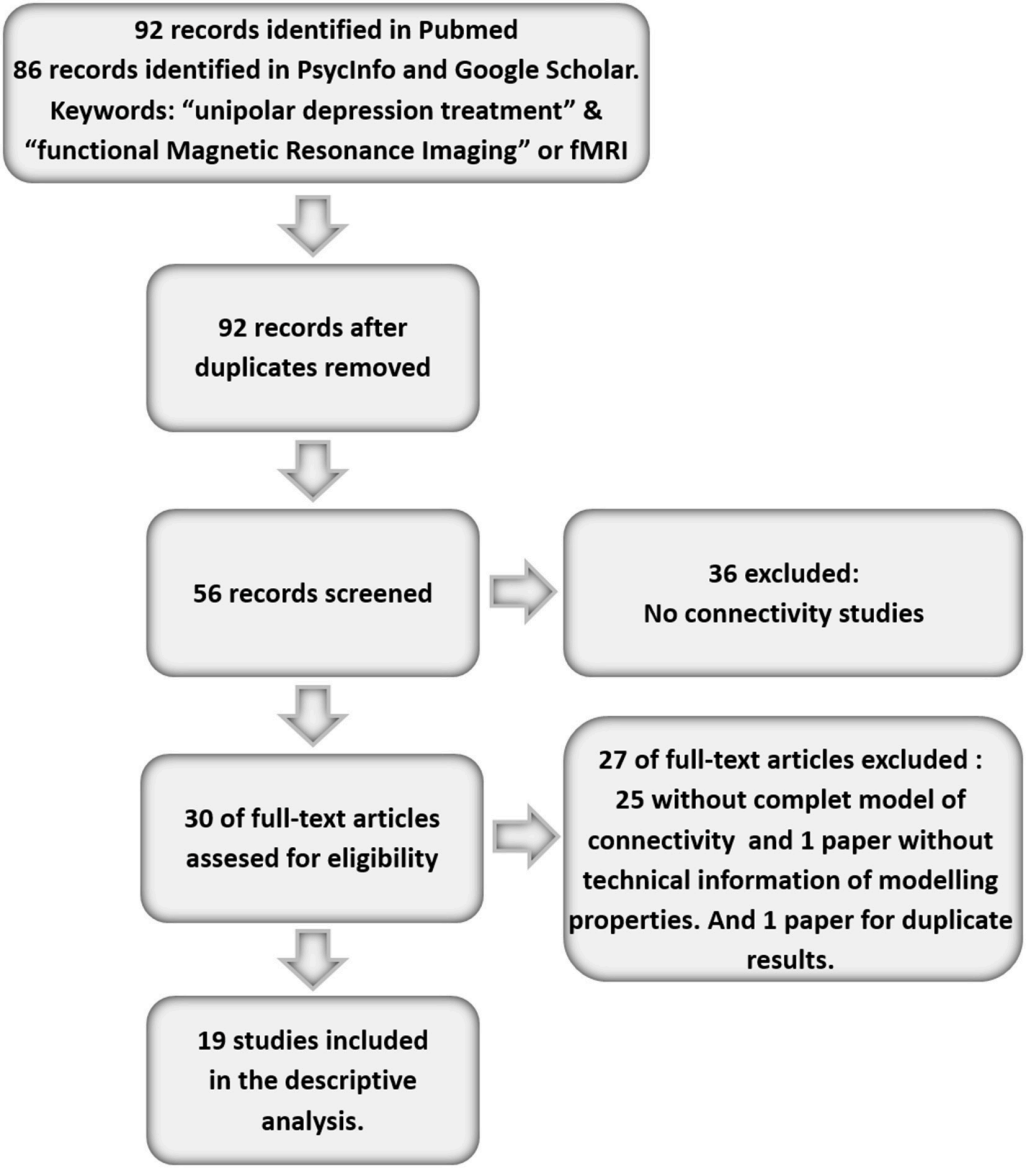
**Flow chart of the paper selection process**.

That same term was also replaced by all the existing second-generation antidepressants, and each antidepressant was combined with the other keywords. Out of all the articles recovered, their suitability was assessed for the goals of the current paper on the base of the selection criteria below. The studies had to contain a sample of patients with one or more active depressive syndromes that had been treated with at least one standard depression treatment: antidepressants, Electroconvulsive Therapy (ECT), or psychotherapy. We also included those treated with the experimental treatment repetitive Transcranial Magnetic Stimulation (rTMS), due to its increasing use. The papers had to take, at least, two fMRI measures in the group of depressed patients, one before and the other after finishing the antidepressive treatment. Under these criteria, and in an early stage, 92 different articles were found in the different databases, once duplicities had been spotted in each database. Out of the articles identified, we deleted those not bearing empirical evidence on one connectivity network linked to MDD. Thirty-six papers were then discarded (39.13%) as they mentioned the topic tangentially. Out of the remaining papers (56), we deleted those that only presented the correlations matrices but not specific connectivity models, given their strictly descriptive goal, regardless of considerations on functional or structural connectivity. Under this criterion, we deleted 26 further papers (28.26%). We analyzed the remaining 30 individually, and we discarded those not offering all the information on the connectivity model, or where such model was partial. This way, we deleted 10 more papers (10.86% of the total), and thus there remained 20 papers suitable for analysis. Eventually, one of them was discarded because the connectivity data were duplicated in a previous publication. Accordingly, there remained a total of 19 articles measuring changes in brain connectivity in depressed patients before and after treatment. The following flow chart shows this selection process of articles analyzed, which carry the symbol ^*^ in the bibliography (Figure 1).

Once the selection process was finished, both researchers conducted the selection of information from each paper and obtained, eventually, a 100% agreement on the data of each paper analyzed.

## Results

After searching and applying the criteria, we found 19 articles measuring changes in brain connectivity associated to antidepressive response (Anand et al., 2005; Chen et al., 2008; Aizenstein et al., 2009; Lisiecka et al., 2011; Wu et al., 2011; Beall et al., 2012; Perrin et al., 2012; Abbott et al., 2013, 2014; Andreescu et al., 2013; Heller et al., 2013; Li et al., 2013; Posner et al., 2013; Baeken et al., 2014; Liston et al., 2014; Salomons et al., 2014; Wang et al., 2014; Wei et al., 2014; Yang et al., 2014).

### Clinical results

Out of the 19 studies analyzed, 11 were conducted on patients with MDD (Anand et al., 2005; Chen et al., 2008; Lisiecka et al., 2011; Beall et al., 2012; Abbott et al., 2013, 2014; Heller et al., 2013; Li et al., 2013; Liston et al., 2014; Salomons et al., 2014; Wang et al., 2014). Three studies were conducted on patients with a MDD diagnosis, but in the Late-onset Depression variant (LOD) (Aizenstein et al., 2009; Wu et al., 2011; Andreescu et al., 2013). One was conducted on dysthymic patients (Posner et al., 2013), one on patients with a “unipolar depression” diagnosis (Yang et al., 2014), two on patients with a “severe depression” diagnosis (Perrin et al., 2012; Baeken et al., 2014), and one on patients with “diagnosis of depression” (Wei et al., 2014). None of the studies were conducted on depressive-stage bipolar patients although one study included 4 patients with that diagnosis in its 21-participant sample (Liston et al., 2014), and another included an unspecified number of bipolar patients out of a total of 6 participants (Beall et al., 2012).

With regard to the treatments administered, 11 studies administered antidepressant treatments (Anand et al., 2005; Chen et al., 2008; Aizenstein et al., 2009; Lisiecka et al., 2011; Wu et al., 2011; Andreescu et al., 2013; Heller et al., 2013; Li et al., 2013; Posner et al., 2013; Wang et al., 2014; Yang et al., 2014). The duration of the treatments varied, ranging from 4 weeks (Lisiecka et al., 2011) to 12 weeks (Aizenstein et al., 2009; Andreescu et al., 2013; Li et al., 2013). In the 11 studies where an antidepressant treatment was administered, it was successful, so the depressive symptoms were reduced or remitted in the majority of the patients. This and further relevant clinical information has been summarized in Table 1.

**Table 1:** **Clinical and demographic characteristics of the studies that addressed the changes in brain connectivity after depression treatment using antidepressants or antidepressants combined with psychotherapy**.

**Study**	**Clinical sample size and age**	**Diagnosis**	**Comorbid illness to depressive syndrome or other characteristics in clinical sample**	**Control sample size and demographic characteristics**	**Treatment**	**Other treatments**	**Depression symptom severity before treatment**	**Depression symptom severity after treatment**
Anand et al., 2005	*n* = 12 mean age 30.0 ± 9.0	MDD	Anxiety disorder comorbid with MDD in some patients	*n* = 12 mean age 29.0 ± 8.0	Sertraline 6 weeks dose range 100–200 mg		HDRS-25 mean 32 ± 8.0	HDRS mean 256.0 ± 6.0
Chen et al., 2008	*n* = 19 mean age 43.3 ± 8.6	MDD	Pure MDD sample	*n* = 19 mean age 42.8 ± 6.7	8-week fluoxetine 20 mg		HDRS mean 21.3 ± 2.4	HDRS 9.3 ± 5.8
Aizenstein et al., 2009	*n* = 13 mean age 69.1 ± 5.79	Late-onset depression	5/13 with Anxiety disorder	*n* = 13 mean age 68.8 ± 5.79	Paroxetine 12 weeks		HDRS mean 19.7 ± 4.2	HDRS mean 7.5 ± 4.8
Lisiecka et al., 2011	*n* = 23 mirtazapine group *n* = 10 mean age 37.7 ± 8.5 venlafaxine *n* = 13 mean age 348.9 ± 9.6	MDD	Some patients had important anxiety symptoms ± HAM-A = 20.29 ± 5.25	None	4-week treatment with either: mirtazapine ± mean dose 37.5 ± 7.9 *n* = 10 venlafaxine mean dose 200 ± 48.9 *n* = 13		Mirtazapine group *n* = 10 HDRS mean 21.6 ± 5.9. Venlafaxine group *n* = 13 HDRS mean 19.5 ± 3.9	Mirtazapine group *n* = 10 HDRS mean 8.5 ± 4.9. Venlafaxine group *n* = 13 HDRS mean 11.6 ± 5.5
Wu et al., 2011	*n* = 8 mean age 70.8 ± 5.7	Late onset depression	Non-psychotic non-bipolar patients	*n* = 12 mean age 69.0 ± 6.5 (final sample *n* = 10)	12-week paroxetine mean dose = 26 ± 11	Weekly interpersonal therapy	HDRS-17 mean 19.8 ± 4.1	HDRS-17 mean = 0.8 ± 4.5
Li et al., 2013	*n* = 16 mean age 32.6 ± 11.84	MDD	Pure MDD sample	*n* = 20 mean age 33.82 ± 10.29	12-week treatment with either: Paroxetine ± 20–60 mg venlafaxine ± 75–225 mg duloxetine ± 60–90 mg or citalopram ± 20–40 mg	7/33 patients receive loracepam ± 5–15 mg during the first 2 weeks of treatment	HDRS-17 mean 26.42 ± 5.22	HDRS-17 mean = 5.13 ± 1.26
Andreescu et al., 2013	*n* = 10 mean age 67.9 ± 4.86	Late onset depression	4/6 recurrent MDD 6/6 single episode	*n* = 47 mean age 72.89 ± 7.90	12-week with: Venlafaxine 210 mg/day *n* = 15. Escitaloram mean dose 15 mg/day *n* = 22. Duloxetine mean dose 100 mg/day *n* = 10.	1 patient augmentation with bupropion	HDRS mean 19.7 ± 4.2	HDRS mean 5.0 ± 2.94
Heller et al., 2013	*n* = 27 mean age 31.48 ± 12.0	MDD	Patients could suffer from comorbid anxiety disorder	*n* = 14 matched to sample characteristics	8 week with: 12/21 fluoxetine 37 mg ± 8.47 or 9/21 treated with venlafaxine 118 mg ± 36.6		HDRS-21 mean 20.6 ± 2.39	9/21 were remitters 6/21 were responders 6/21 were non-responders
Posner et al., 2013	*n* = 41 ± 32 finished the trial mean age = 37.8 ± 9.0	Dystimic disorder without current MDD episode	Patients could suffer from comorbid anxiety disorder	*n* = 25 mean age 33.0 ± 11.9	10-week of either duloxetine 30–120 mg or placebo	Placebo group treated with placebo	Duloxetine treated patients HDRS-24 mean 20.0 ± 0.9. Placebo treated patients HDRS-24 mean 21.4 ± 0.8	Duloxetine treated patients HDRS-24 mean 5.8 ± 1.6. Placebo treated patients HDRS-24 mean 17.3 ± 1.5
Wang et al., 2014	*n* = 20 mean age 34.6 ± 12.2	MDD	Pure MDD sample	*n* = 20 mean age 34.7 ± 12.2	8-week escitalopram 10–30 mg		HDRS-17 mean 27.9 ± 4.0	HDRS-17 mean 7.4 ± 6.7
Yang et al., 2014	*n* = 12.3 mean age 4.091 ± 12.6 Final sample *n* = 10	Unipolar depression	Pure depressed patients	None	8-week sertraline 50–100 mg		HDRS mean 24.08 ± 4.40	HDRS mean 5.83 ± 2.32

*n, sample size; MDD, Major depressive disorder; MADRS, Montgomery Adsberg Depression Scale; HDRS, Hamilton Depression Rating Scale. The number following the acronym HDRS indicates the number of items of the particular version of the scale used*.

Out of the 18 papers analyzed, 5 of them studied connectivity after ECT (Beall et al., 2012; Perrin et al., 2012; Abbott et al., 2013, 2014; Wei et al., 2014). The treatment was administered in a number of sessions ranging from an average of 7 (Wei et al., 2014) to 11 (Abbott et al., 2013, 2014). All the papers with low numbers show that the treatment was administered efficiently, and that the response or remission of depressive symptoms was achieved in the majority of patients. Three papers used rTMS as a depression treatment (Baeken et al., 2014; Liston et al., 2014; Salomons et al., 2014). In these papers, 25 and 20 rTMS sessions were administered, respectively. All the papers obtained a response or a partial response to such antidepressive therapy. This and further relevant clinical information has been summarized in Table 2.

**Table 2:** **Clinical and demographic characteristics of the studies that addressed the changes in brain connectivity after depression treatment using Electroconvulsive Therapy (ECT) and repetitive Transcranial Magnetic Stimulation (rTMS)**.

**Study**	**Clinical sample size and demographic characteristics**	**Diagnosis**	**Comorbid conditions to depressive illness or other characteristics in clinical sample**	**Control sample size and demographic characteristics**	**Treatment**	**Other treatments**	**Depression symptom severity before treatment**	**Depression symptom severity after treatment**
Perrin et al., 2012	*n* = 9 mean age 46.8 ± no data	Severe depression	Not specified	None	8.3 ± no data ECT sessions		MADRS mean 36.4 ± 4.9	MADRS = 36. ± 44.9
Beall et al., 2012	*n* = 6.39.0 ± 5.4	MDD	MDD and Bipolar patients in the sample; 6/6 treatment resistant	None	8.8 ± 3.2 ECT sessions		HDRS = 25.17 ± 4.71	HDRS-17 = 9.33 ± 3.6
Abbott et al., 2013	*n* = 12. mean age 66.4 ± 9.78	MDD	T3/12 MDD subjects had psychotic symptoms	*n* = 12. mean age 67.58 ± 8.48	11.17 ± 3.33 ECT sessions	12/12 treated with antidepressants. 8/12 treated with antipsychotics. 1/12 treated with mood stabilizers	Remitters *n* = 9 HDRS-24 mean 34.56 ± 10.2 Non-Remitters *n* = 3 HDRS- 24 mean 33.67 ± 6.66	Remitters *n* = 9 HDRS-24 = 2.89 ± 2.93 Non-Remitters *n* = 3 HDRS-24 = 18.33 ± 3.51
Abbott et al., 2014	*n* = 15. mean age 65.5 ± 6.70	MDD	11/19 MDD subjects had psychotic symptoms	*n* = 20. mean age 64.90 ± 9.40	11 ± 2.7 ECT sessions	19/19 treated with antidepressants. 11/19 treated with antipsychotics	HDRS-24 mean 32.6 ± 8.5	HDRS-24 8.4 ± 8.6
Liston et al., 2014	*n* = 17 mean age 42.3 ± 17.3	MDD or bipolar type II disorder	Treatment resistant patients	*n* = 35 mean age 36.0 ± 16.0	25 sessions of 10-hz TMS during 5-week period	All patients treated with antidepressants	Not specified	HDRS improved mean = 9.1 ± 7.5
Salomons et al., 2014	*n* = 25 mean age 42.6 (range 19–70)	MDD (*n* = 21) bipolar Type 1 (*n* = 1) or bipolar Type II (*n* = 3)	Treatment-resistant patients	None	20 sessions of 10-hz rTMS to the bilateral dmPFC during 4-week-period		HDRS-17 mean 21.3 ± 6.7	HDRS-17 = 12.0 ± 8.2
Baeken et al., 2014	*N* = 5 40.0 ± 13	Severe unipolar depression	Treatment-resistant patients	None	20 High Frequency rTMS sessions during 1 week	All patients antidepressant-free	HDRS-17 mean 18 ± 9	HDRS-17 = 8 ± 2
Wei et al., 2014	*n* = 11	Depression	11/11 treatment resistant.	*n* = 15 matched to clinical sample for age gender and education years	6.82 ± 2.40 bifrontal ECT sessions	11/11 treated with antidepressants 3/11 treated with antipsychotics. 1/11 with lithium	HDRS-17 mean 21.91 ± 4.15	HAM-D1-7 = 13.91 ± 2.39

*n, sample size; MDD, Major depressive disorder; MADRS, Montgomery Adsberg Depression Scale; HDRS, Hamilton Depression Rating Scale. The number following the acronym HDRS indicates the number of items of the particular version of the scale used*.

### Connectivity results

Out of the 18 studies, 12 had a “longitudinal case-control study” design (Anand et al., 2005; Chen et al., 2008; Aizenstein et al., 2009; Wu et al., 2011; Abbott et al., 2013, 2014; Andreescu et al., 2013; Heller et al., 2013; Li et al., 2013; Liston et al., 2014; Wang et al., 2014; Wei et al., 2014). Out of these, however, the paper by Heller et al. (2013) reports pre-post results only for the behavioral data. One paper had a Mixed-factorial design with two treatment groups (Lisiecka et al., 2011). One combined a mixed factorial design with placebo-controlled clinical trial methodologies (Posner et al., 2013), and one had a sham-controlled cross-over design (Baeken et al., 2014). The remaining works were conducted with a pre- and post-treatment design (Beall et al., 2012; Perrin et al., 2012; Salomons et al., 2014; Yang et al., 2014). According to the type of paradigm, out of the 11 papers that administered antidepressant treatments, 6 assessed connectivity at rest (Wu et al., 2011; Andreescu et al., 2013; Li et al., 2013; Posner et al., 2013; Wang et al., 2014; Yang et al., 2014), 4 assessed brain connectivity using different activation paradigms (Chen et al., 2008; Aizenstein et al., 2009; Lisiecka et al., 2011; Heller et al., 2013), and one work used both approaches (Anand et al., 2005). Out of the 8 works that treated patients with somatic non-pharmacological treatments, 7 were performed at rest (Perrin et al., 2012; Abbott et al., 2013, 2014; Baeken et al., 2014; Liston et al., 2014; Salomons et al., 2014; Wei et al., 2014) and one paper used emotional and cognitive paradigms and resting measures (Beall et al., 2012).

These and further methodological characteristics have been summarized in Tables 3A,B, 4A,B.

**Table 3A:** **Connectivity results in works that measured connectivity at rest and that treated their patients with antidepressants or antidepressants combined with psychotherapy**.

**Study**	**Study design**	**fMRi protocol design**	**Data analysis**	**Main findings before treatment**	**Main findings after treatment**	**Correlation between connectivity changes and symptoms improvement**	**Other findings**
Wu et al., 2011	Longitudinal case control study	Slow-frequency finger tapping^*^	Seed-based: seed at PCC	LOD < HC PCC with sgACC (BA 25). LOD > HC PCC with dmPFC (BA 6) and OFC.	↑ PCC with sgACC and mPFC. Lower activations in sgACC and higher activations in rostral ACC and dorsal ACC	Analysis not performed	Pre-treatment higher white matter intensities volume was highly associated with lower connectivity
Li et al., 2013	Longitudinal case control study	Rest with eyes closed	ICA with DMN template	MDD > HC in: bilateral pre-cuneus of the posterior sub-network of the DMN and MPFC of the anterior sub-network of the DMN	MDD = HC in bilateral pre-cuneus connectivity. No normalization of hiperconnectivity in mPFC of MDD patients	No association	
Andreescu et al., 2013	Longitudinal case control study	Rest viewing a fixation point	Seed based. Seed at PCC comparisons with other DMN structures	LOD > HC ↑ PCC- Precuneuus, L_insula, and L_HPC	↑ Medial frontal Gyrus and dACC	Analysis not performed	
Posner et al., 2013	Longitudinal case control study and placebo-controlled clinical trial	Rest with eyes closed	Seed based: seed at PCC	MDD > HC PCC with: medial prefontal cortex, lateral parietal lobes, and pre-cuneus. MDD had greater DMN connection density than HC	Duloxetrine treated patients: ↓ PCC with: right lateral parietal cortex, right mid-superior frontal cortex, and right inferior temporal gyrus. Reduction in the DMN density. No connectivity changes in placebo group.	No association	
Wang et al., 2014	Longitudinal case control study	Rest with eyes closed	Whole brain analysis. Functional connectivity strength maps (FCS). Seed-based analysis with seeds at dmPFC and HPC	MDD < HC on FCS left superior temporal gyrus, right angular gyrus, and occipital regions. MDD > HC on FCS in the right medial frontal gyrus, right supplemental motor area, and right parahipoccampal gyrus. MDD > HC in dmPFC with: lSFG, l_ dmPFC, and bilateral thalamus. MDD < HC HPC with cerebellum	FCS in bilateral dmPFC reduced. FCS↑ between hippocampi and left cerebellum. ↓ dmPFC with: lSFG, left dmPFC, and thalamus. ↑ between hippocampi and cerebellum	FCS and Resting connectivity changes in dmPFC positively correlated with changes in HDRS.	
Yang et al., 2014	Pre-treatment–post-treatment	Rest	Seed based: seed at hypotalamus	None	↑ HPT with: DLPFC, OFC, SFG, pre-central gyrus, ACC, HPC, putamen insula, and claustrum. And areas of temporal and parietal lobules ↓ HPT with: IFG, MFG, SFG, cingulate gyrus, MTG, pre-cuneus, thalamus, and cerebellum		

*BA, Brodmann area; ACC, Anterior cingulate cortex; MPFC, medial prefrontal frontal cortex; IFG, inferior Frontal gyrus; MFG, Middle Frontal Gyrus; SFG, superior frontal gyrus; PCC, Posterior cingulate cortex; DLPFC, dorsolateral prefrontal cortex; OFC, Orbitofrontal Cortex; MTG, Middle Temporal Gyrus; l_MTL, medial temporal lobe; HPC, Left hippocampus; HPT, Hypothalamus; ICA, independent component analysis; DMN, Default Mode Network; d, dorsal; Dm, Dorsomedial; Sg, Subgenual; Pg, Pregenual; L, Left; FCS, functional connectivity strength; <, Lower connectivity than; >, Higher connectivity than; ↑, increased connectivity; ↓, Decreased connectivity; MDD, major depressive disorder; LOD, Late onset Depression; HC, healthy controls; <, Lower connectivity than; >, Higher connectivity than; ↑, increased connectivity; ↓, Decreased connectivity; NDD, major depressive disorder; HC, healthy controls*.

* *Simple sensory motor tasks such as slow-frequency finger tapping do not interfere with DMN activity and have been used in the literature to obtain resting-state data (Greicius et al., 2003)*.

**Table 3B:** **Connectivity results in works that measured connectivity by using task-paradigms and that treated their patients with antidepressants or antidepressants combined with psychotherapy**.

**Study**	**Study design**	**fMRi protocol design**	**Data analysis**	**Main findings before treatment**	**Main findings after treatment**	**Correlation between connectivity changes and symptoms improvement**	**Other findings**
Anand et al., 2005	Longitudinal case control study	Passive affective task. Rest with eyes closed prior to the task	Seed to voxel correlation between ACC and: MTHAL, PST and AMY	MDD < HC ACC-MTHAL, ACC-pallidostriatum with positive pictures, ACC-l_MTHAL, ACC-PST with negative pictures	MDD = HC at rest and on exposure to neutral positive and negative stimuli. MDD ↑ at rest ACC-rMTHAL and ACC-LMTHAL. ↑ ACC-MTHAL on exposure to neutral and positive stimuli	Association between ↑ ACC-l_AMY for neutral pictures and decrease in HDRS	
Chen et al., 2008	Longitudinal case control study	Sad facial processing task	Seed based seeds at AMY	MDD < HC, AMY with: HPC, AMY, putamen, insula parahipoccampal gyrus, temporal cortices, inferior, and medial frontal cortex	MDD = HC in AMY connectivity. MDD had greater time-related ↑ AMY with-r mPFC, ACC, insula, thalamus, caudate nucleus, and putamen	Analysis not performed	
Lisiecka et al., 2011	Mixed factorial design with two treatment arms	Human emotional expressions recognition task	Seed based. Seed at OFC whole brain correlation with bilateral OFC	None	↑ Connectivity between OFC and: right crebellum, right pre-cuneus, left middle cingulate cortex, and left superior parietal gyrus (including pre-cuneus). ↓ OFC and: r MCC, MTG, superior occipital gyrus, right fusiform gyrus and inferior temporal gyrus. ↓ l_OFC connectivity with: left superior parietal gyrus, pre-cuneus and post-central gyrus. Left MTG, Cuneus, calcarine fissure and angular gyrus. ↑ OFC coupling Responders > non -responders between OFC and: Gyrus rectus, right caudate, thalamus, SMA. ↑ OFC coupling responders > non responders in l_MCC left paracentral gyrus and cerebellum	Analysis not performed	In patients treated with venlafaxine: ↑ OFC connectivity with: right cerebellum, right pre-cuneus. ↓ OFC connectivity with right post-central and precentral giry, rMCC, pre-cuneus, cuneus superior occipital gyrus, right lingual gyrus, vermis, cerebellar areas and inferior temporal gyrus. In mirtazapine treated patient's ↑ OFC connectivity with: r middle Frontal gyrus. ↓ In OFC connectivity with: r_MTG, Angular gyrus, r_MCC, superior occipital gyrus, right fusiform gyrus, and l_MTG
Heller et al., 2013	Pre-and post-treatment study	Task of positive and negative affective processing with enhancement of positive emotions	Seed based: seeds at nucleus accumbens	None	↑ sustained connectivity between accumbens-MFG, including BA 46 and BA 10 associated to gains in affect. ↑ sustained connectivity between accumbens-MFG. ↑ aggregated connectivity Accumbens-VMPFC	Several correlations between changes in connectivity and increases in positive affect	Correlation between change in sustained connectivity Accumbens-BA46 and positive affect not differ between two medication groups
Aizenstein et al., 2009	Longitudinal case control study	POP task (cognitive control task)	Seed based at: DLPFC (BA 9 and 46) dACC	LOD had lower correlations between dACC and dLPFC than HC	No changes in connectivity dACC-DLPFC		

*MTHAL, medial thalamus; ACC, Anterior cingulate cortex; PST, Pallidostriatum; AMY, amygdala DLPFC, dorsolateral prefrontal cortex; BA, Brodmann area; HPC, Hippocampus; OFC, Orbitofrontal Cortex; MPFC, medial prefrontal frontal cortex; MCC, middle cingulated cortex; MTG, medial temporal gyrus; SMA, supplementary motor area; VMPFC, ventromedial prefrontal cortex; MFG, Middle Frontal Gyrus; SFG, superior frontal gyrus; l, left; r, Right; <, Lower connectivity than; >, Higher connectivity than ↑, increased connectivity; ↓, Decreased connectivity; MDD, major depressive disorder; LOD, Late onset depression; HC, healthy controls*.

**Table 4A:** **Connectivity results in works that measured connectivity at rest and treated their patients with ECT therapy or TMS therapy**.

	**Study design**	**fMRi protocol design**	**Data analysis**	**Main findings before treatment**	**Main findings after treatment**	**correlation between connectivity changes and symptoms improvement**	**Other findings**
Perrin et al., 2012	Pre- and post-treatment design	Virtual Ball Passing task^*^	Whole Brain connectivity data driven CHART analysis	None	↓in voxels within and around l_DLPFC (including BA 44, 45 and 46). ↓ between ACC MPFC and l-DLPFC with l_SMG, l-AG and somatosensory association cortex	Analysis not conducted	
Abbott et al., 2013	Longitudinal case control study	Rest viewing a fixation point	Spatial ICA and components of interest: a_DMN, p_DMN, DLPFC, dmPFC dmP	MDD < HC in p_DM with DMPFC and p_DMN and l_DLPFC	In MDD remitters FC between p_DMN and dmPFC changed from − to +; FC between p_DMN and l_DLPFC changed from − to +; FCN post ETC in MDD remitters = HC	Not found	
Abbott et al., 2014	Longitudinal case control study	Rest viewing a fixation point	Seed based: seds at l_HPC and r_HPC.	MDD < HC in: rHC with LMTL and ACC-l_HPC with rMTL	↑rHC. HC Connectivity maps MDD = HC	Moderate correlations between clinical improvement and changes in connectivity	RHC volume increased with treatment. This finding correlates with clinical improvement
Liston et al., 2014	Longitudinal case control study	Rest	Seed based. Seeds at DLPFC and sgACC Targets in the CEN and DMN. Generation of two within-network connectivity maps: DLPFC-CEN sgACC-DMN and two between network maps: DLPFC-DMN and sgACC-CEN	Within the CEN: MDD < HC between lDLPFC and: premotor cortex (BA 46), posterior parietal areas (BA 40 BA 47), bilateral cerebellum and lateral prefrontal cortex (BA 8, 9). MDD >HC within the DMN: ↑ sgACC and: vmPFC, pgACC, thalamus and pre-cuneus	Connectivity reductions DLPFC-CEN persisted. ↓sgACC-DMN	Analysis not performed	
Salomons et al., 2014	Pre- and post-treatment design	Rest with eyes closed	Seed based: two seeds at dmPFC: one above the Cingulate cortex, one in anterior midcingulate cortex (aMCC). Seed at sgACC	None	↑ dmPFC with thalamus. ↓ dmPFC with: bilateral insula, parahippocampal gyrus and amygdala. ↓sgACC with ventral striatum and dmPFC associated with better response to treatment	↑ dmPFC and thalamus significantly correlated with HDRS improvement. ↓sgACC with caudate correlated with HDRS improvement	
Baeken et al., 2014	Sham-controlled cross-over design	Rest with eyes closed	Seed based: seed at sgACC	None	↑sgACC and perigenual ACC	Analysis not conducted	
Wei et al., 2014	Longitudinal case control study	Rest with eyes closed	Voxel Mirrored Homotopic Connectivity (VMHC)	MDD < HC VMHC between Frontal gyri (BA 8/9) angular gyri (BA 39)	↑ VMHC between both MFG (BA8) and both SFG (BA 10).WMHC MDD = HC between: The middle frontal gyri (BA8/9), angular gyri (BA 39)	Not found	

*DLPFC, dorsolateral prefrontal cortex; BA, Brodmann area; SMG, supramrginal gyrus; AG, angular Gyrus; P_DMN, Posterior Default mode Network; a_DMN, anterior Default Mode Network; dmPFC, Dorsomedial Prefronal cortex; HPC, Hippocampus; ACC, Anterior cingulate cortex; CEN, control Executive Network; DMN, default mode Network; vmPFC, ventromedial prefrontal cortex; MCC, Middle cingulated cortex; VMHC, voxel Mirrored Homotopic Connectivity; a, anterior; Sg, subgenual; Pg, pregenual; Dm, dorsomedial; Vm, ventromedial; l, left; r, Right; <, Lesser connectivity than; >, Higher connectivity than; ↑, increased connectivity; ↓, Decreased connectivity; MDD, major depressive disorder; HC, healthy control*.

* *simple sensory motor tasks such as slow-frequency finger tapping do not interfere with DMN activity and have been used in the literature to obtain resting-state data (Greicius et al., 2003)*.

**Table 4B:** **Connectivity results in works that measured connectivity during task-paradigms and that treated their patients with with ECT therapy or TMS therapy**.

**Study**	**Study design**	**fMRI protocol design**	**Data analysis**	**Main findings before treatment**	**Main findings after treatment**	**correlation between connectivity changes and symptoms improvement**	**Other findings**
(Beall et al., 2012)	Pre- and post-treatment design	Working Memory Task; Affective Task: neutral and unpleasant pictures viewing. Rest with eyes closed	Seed based. Seeds at: ACC. MPFC, r_DLPFC, s_PL, I_PL. PCC. Superior Cerebellum. OFC.PMC	None	↑ Between r_ACC- OFC and ACC-caudate. ↓ Between l_ACC - OFC. ↑ Between ACC - r_DLPFC and ACC -PCC	Association between clinical improvement and changes in connectivity values from r-ACC and r-DLPFC	

*R_DLPFC, right dorsolateral prefrontal cortex; L_DLPFC, left dorsolateral prefrontal cortex; BA, Brodmann area; ACC, Anterior cingulate cortex; MPFC, medial prefrontal frontal cortex; PCC, Posterior cingulate cortex; OFC, Orbitofrontal Cortex; <, Lower connectivity than; >, Higher connectivity than; ↑, increased connectivity; ↓, Decreased connectivity*.

As regards the studies' results on connectivity, seven studies approached the effect of depression treatment on DMN connectivity (Wu et al., 2011; Beall et al., 2012; Abbott et al., 2013; Andreescu et al., 2013; Li et al., 2013; Posner et al., 2013; Liston et al., 2014). Four studies (Andreescu et al., 2013; Li et al., 2013; Posner et al., 2013; Liston et al., 2014) found DMN hyperconnectivity in depressed patients with respect to the control subjects. In all of them, connectivity reductions were observed in DMN after the treatment (Li et al., 2013; Posner et al., 2013; Liston et al., 2014), except for the paper by Andreescu et al. (2013), where—somewhat paradoxically—it was associated to an increased connectivity in the anterior, frontal nodes of the DMN, in which such “frontalization” is interpreted as a possible normalizing effect of antidepressant treatment. The paper by Abbott et al. (2013) suggested that patients with MDD present a hyperconnectivity pattern in the posterior areas of DMN, PFDLC, and dmCPF, which tends to recover after the treatment. The paper by Wu et al. (2011) showed a mixed pattern of hypoconnectivity in the posterior areas of DMN, and hyperconnectivity in the most anterior areas of the same network at rest in depressed patients. In the same paper, hypoconnectivity between PCC and sgACC recovered, but not PCC-dmPFC hyperconnectivity (Wu et al., 2011). The paper by Beall et al. (2012), with a pre- and post-treatment design, showed an increase in PCC-ACC connectivity after ECT treatment. Four studies investigated the possible correlation between clinical improvement of depression and changes in connectivity (Beall et al., 2012; Abbott et al., 2013; Li et al., 2013; Posner et al., 2013). Only the study by Beall et al. (2012) found that clinical improvement correlated with changes in ACC-DLPFC connectivity. The other papers found no correlations between clinical symptom improvement and changes in connectivity (Abbott et al., 2013; Li et al., 2013; Posner et al., 2013). The specific results from each study have been summarized in Tables 3A,B, 4A,B.

Six studies approached the changes in connectivity after depression treatment in cortical-limbic connectivity, one of them conducted at rest (Salomons et al., 2014), and the others using several affective tasks (Anand et al., 2005; Chen et al., 2008; Lisiecka et al., 2011; Beall et al., 2012; Heller et al., 2013). Two of them compared the connectivity between ACC, frontal and limbic regions of depressed patients with control patients at the baseline measure. Both presented similar results. On the one hand, the one by Anand et al. (2005) found hypoconnectivity between ACC and the subcortical structures such as the medial thalamus and the pallidostriatum. On the other, Chen et al. (2008) found a pattern of hypoconnectivity between the amygdala and several cortical and subcortical structures (Chen et al., 2008). Likewise, both papers found important increases in connectivity after the treatment. In the paper by Anand et al. (2005) no differences in connectivity were found after the treatment between the patients and the control subjects at rest and when exposed to negative stimuli (visual images with negative affective valence from “The International Affective Picture System”; IAPS). It has also been suggested that the amygdala connectivity becomes normalized after antidepressive treatment (Chen et al., 2008). The paper by Lisiecka et al. (2011) compared the changes in brain connectivity between two groups of depressed patients, one treated with venlafaxine and the other with mirtazapine. After the treatment, the patients presented increases in connectivity between dmPFC and cerebellum, cingulated cortex, parietal cortex, and decreases in connectivity between the OFC and the right medial cingulated cortex, the middle temporal gyrus, the superior occipital gyrus, the right fusiform gyrus, and the inferior temporal gyrus. Also the same paper showed decreases in connectivity between left the OFC and the left superior parietal gyrus, the pre-cuneus, and the post-central gyrus, the left medial temporal gyrus, the cuneus, the calcarine fissure, and the angular gyrus (Lisiecka et al., 2011). This study also suggested that venlafaxine changed the OFC-cerebellum coupling, whereas mirtazapine increased the OFC-DLPFC connectivity. Three studies measured changes in brain connectivity after antidepressant treatment in a pre- and post-treatment design (Beall et al., 2012; Heller et al., 2013; Salomons et al., 2014). The work by Beall et al. (2012) found a pattern of increases and decreases in connectivity between ACC, the caudate nucleus and frontal and parietal cortical areas. The work by Salomons et al. (2014) showed—among other findings—decreases in connectivity between dmPFC and some limbic structures. The work by Heller et al. (2013) found connectivity between the nucleus accumbens, MFG and accumbens-VMPFC related to changes in affection. Out of all these papers, four of them (Anand et al., 2007; Beall et al., 2012; Heller et al., 2013; Salomons et al., 2014) conducted analyses of the correlations between connectivity changes and the improvement of depressive symptoms measured through clinical scales (Anand et al., 2007; Beall et al., 2012; Salomons et al., 2014), or correlations between changes in connectivity and specific symptoms improvement, such as alterations of affection or anhedonia (Heller et al., 2013). The four papers found a positive link between both types of variables. Each paper's specific results have been summarized in Tables 3A,B, 4A,B.

Three papers studied changes in connectivity in CCN after depression treatment (Aizenstein et al., 2009; Perrin et al., 2012; Liston et al., 2014). In the first paper, the authors found that depressed patients showed less connectivity between DLPFC and dACC than control subjects (Aizenstein et al., 2009). The paper by Liston et al. (2014) found hypoconnectivity in depressed patients between DLPFC and MFG (BA 46), posterior parietal areas, and other prefrontal areas (Liston et al., 2014). In none of the studies was connectivity recovered after depression treatment (Aizenstein et al., 2009; Liston et al., 2014), although the study by Liston et al. (2014) did find improvement after treatment in DMN. The paper by Perrin et al. (2012) suggested that patients experienced reductions in connectivity related to the treatment within l_DLPFC and the prefrontal cortex (BA 44, 45, and 46) as well as reductions in connectivity between ACC and areas of OFC and r-DLPFC among others (Perrin et al., 2012). Each paper's specific results have been summarized in Tables 3A,B, 4A,B.

Lastly, six papers could not be classified into the above division. The paper by Abbott et al. (2014) suggested that depressed patients show hypoconnectivity of each hippocampus with the contralateral medial temporal lobe and of r_HPC with ACC. In this study, after the treatment, only the right hippocampus connectivity increased, which the authors attribute to the hemisphere where ECT was applied (Abbott et al., 2014). Moderate correlations were reported in this paper between connectivity recovery and clinical improvement. On the other hand, the paper by Wei et al. (2014) suggested that depressed patients present hypoconnectivity in the Voxel Mirrored Homotopic Connectivity between both MFG (BA 8/9) and both angular gyri (BA 49). The paper also suggested that, after treatment, the connectivity between both hemispheres increases and tends to become normalized (Wei et al., 2014). The paper by Wang et al. (2014) suggested that depressed patients show smaller Functional Connectivity Strength maps (FCS) than healthy subjects in both hippocampi, and the opposite pattern in the dmPFC cortexes of both hemispheres. Antidepressant treatment tended to reverse the pattern so that, after treatment, FCS increased between both hippocampi and it decreased between dmPFCs. Additionally, the changes in dmPFC connectivity correlated positively with the clinical improvement measures (Wang et al., 2014). In the paper by Yang et al. (2014), we observed increases in the connectivity of the hypothalamus with temporal areas and of the basal ganglia, and decreases of the connectivity of the hypothalamus with frontal and medial areas, the thalamus, and the cerebellum (Yang et al., 2014). The work by Baeken et al. (2014) found that the successful depression treatment with high frequency rTMS was associated to an increase in connectivity between sgACC and perigenual ACC, both seeds of either DMN and AN. Each paper's specific results are summarized in Tables 3A,B, 4A,B.

## Discussion

The goal of the current paper is to review the studies investigating the effect of antidepressant treatment on brain connectivity. Some conclusions may be extracted from the current review. Firstly, out of the 19 papers reviewed, all but one (Aizenstein et al., 2009) reported that, after depression treatment and the corresponding improvement, there occur changes in brain connectivity. As can be observed in Tables 1, 2, despite the fact that most of the studies were conducted on patients with MDD (Anand et al., 2005; Chen et al., 2008; Lisiecka et al., 2011; Beall et al., 2012; Abbott et al., 2013, 2014; Heller et al., 2013; Li et al., 2013; Liston et al., 2014; Salomons et al., 2014; Wang et al., 2014), depression treatment is also related to changes in connectivity in patients with LOD (Wu et al., 2011; Andreescu et al., 2013), in dysthymic patients (Posner et al., 2013), and in more heterogenic samples of patients with unipolar depression (Perrin et al., 2012; Wei et al., 2014; Yang et al., 2014). Likewise, several of these papers include, among their samples, treatment-resistant patients, or ones with comorbid anxiety disorders among other clinical characteristics. Accordingly, successful treatment of unipolar depression seems to be generally related to changes in brain connectivity.

As regards the connectivity networks studied in the current paper, the results summarized in Tables 3A,B, 4A,B allow us to conclude that both depression and depression improvement are related to changes in connectivity in DMN as all the papers included in this review find this association positive (Wu et al., 2011; Beall et al., 2012; Abbott et al., 2013; Andreescu et al., 2013; Li et al., 2013; Posner et al., 2013; Liston et al., 2014). Most of these papers found basal a hyperconnectivity pattern in the DMN of depressed patients—already discussed in the Results Section—except for the paper by Abbott et al. (2014), where a different pattern was found. This hyperconnectivity pattern in DMN is to be expected given that—as discussed in the introduction—it is a common finding in most studies and was confirmed in a recent meta-analysis (Kaiser et al., 2015). It is an interesting fact that all the studies reviewed found changes in DMN connectivity after treatment, and most studies found that DMN connectivity tends to normalize or resemble more that of healthy subjects after treatment (Wu et al., 2011; Abbott et al., 2013; Andreescu et al., 2013; Li et al., 2013; Posner et al., 2013; Liston et al., 2014). However, most studies—except for Abbott et al. (2013) maybe—suggested that the normalization of DMN connectivity after treatment is incomplete. In the paper by Andreescu et al. (2013), the antidepressant treatment was associated to an increased connectivity in the anterior frontal nodes of the DMN, but the alterations of PCC connectivity found in their depressed patients prior to treatment was not recovered. In the paper by Liston et al. (2014), the treatment was associated to a decrease in sgACC connectivity with other DMN structures, but the connectivity of pgACC with the thalamus and the pre-cuneus did not change.

Something similar may be concluded from the studies measuring connectivity changes between cortical-limbic structures (Anand et al., 2005; Chen et al., 2008; Lisiecka et al., 2011; Beall et al., 2012; Heller et al., 2013; Salomons et al., 2014). In fact, in all these papers, the patients showed changes in connectivity between different cortical and limbic structures after depression treatment. According to the emotional valence of the stimulus, the papers by Anand et al. (2005) and Heller et al. (2009) showed similar results given that, in both papers, structures that are critical to emotional processing such as ACC and AMY present hypoconnectivity in depressed patients when compared to healthy ones in situations of negative emotions processing. More importantly, their data suggests that antidepressant treatment tends to normalize the connectivity in these structures (Anand et al., 2005; Chen et al., 2008). The paper by Beall et al. (2012) showed that ECT treatment diminished the activity of OFC when faced with negative valence stimuli. Nevertheless, the connectivity analysis of this paper was made only for the rest condition, and the authors found the hyper and hypoconnectivity mixed pattern of the OFC that is described in Table 4B (Beall et al., 2012), which is difficult to interpret due to the methodology used in that study. The paper by Heller et al. (2013) focuses on positive emotional processing. A previous study not included in this meta-analysis—(Heller et al., 2009)—showed that depressed patients had difficulty in sustaining the connectivity between the nucleus accumbens and the DLPFC when they were instructed to enhance positive emotions elicited by positive images. In the paper included in our review, its data suggests that antidepressant treatments improved sustained connectivity between the nucleus accumbens and vmPFC when patients performed the same kind of task, and these gains were related to gains in positive affect after treatment (Heller et al., 2013). The work by Lisiecka et al. (2011) is difficult to discuss in terms of the emotional valence of the stimulus because the authors performed an OFC seed-whole brain connectivity analysis and found a very complex pattern of OFC connectivity changes that is hardly interpretable in terms of functional connectivity related to the emotional valence processing. Finally the paper by Salomons et al. (2014) showed that a successful treatment was associated with increased dmPFC-thalamic connectivity and decreased sgACC-caudate connectivity. Although performed at rest, it should be noted that, in their study, these changes strongly correlated with improvements in depressive symptoms. In other studies, sgACC connectivity with striatal regions has been related to the maladaptive emotion regulation of depressed patients (Furman et al., 2011). Therefore, the connectivity changes in the paper by Salomons et al. (2014) were possibly somewhat related to changes in positive or negative emotional processing. Taken together, the results of our review resembles to some extent those of Ma (2014), because successful antidepressant treatment may be associated to both activation and connectivity changes in different emotional sub-networks during emotional negative processing, and probably also during positive emotional experiences. However, the number of connectivity studies performed to date is too small to establish the specific connectivity modifications after depression treatment.

Nonetheless, it is not clear that the same thing happens with the connectivity of the Cognitive Network structures since, out of the three papers studying this network (Aizenstein et al., 2009; Perrin et al., 2012; Liston et al., 2014), only one reports a positive relationship between connectivity changes and depression improvement (Perrin et al., 2012), whereas longitudinal-case control studies show more permanent alterations in this network. Although by no means does this allow us to conclude that CCN is a treatment-resistant network, it is noteworthy that it is the only network yielding this type of result. It is even more noteworthy if we consider that, in addition to the papers on DMN and cortical-limbic connections, there exist reports on the positive relationship between depression improvement and connectivity changes in the hippocampus (Abbott et al., 2013), the hypothalamus (Yang et al., 2014), between both brain hemispheres (Wei et al., 2014), between sgACC and perigenual ACC (Baeken et al., 2014), and in studies conducted with connectivity analyses of the whole brain (Wang et al., 2014). Undoubtedly, further studies are needed to find out what happens to CCN after treatment.

We find positive correlations between clinical improvement and changes in brain connectivity in three papers investigating cortical-limbic connectivity changes (Anand et al., 2007; Heller et al., 2013; Salomons et al., 2014): one investigates connectivity changes between HPC and dmPFC and the rest of the brain (Wang et al., 2014); and then the paper by Beall et al. (2012), which was conducted with seeds belonging to DMN and the limbic system. However, the papers investigating the relationship between depression treatment and connectivity changes in DMN found no such association (Abbott et al., 2013; Li et al., 2013; Posner et al., 2013). Taken together, the data suggest the possibility of a relationship between recovery from depression symptoms and recovery of connectivity between several cortical-limbic structures. The close relationship between clinical recovery and connectivity changes between these brain areas may be somewhat expectable because the limbic system is closely related to the mood, anxiety, and vegetative symptoms that constitute the core depressive symptoms. However, the lack of a relationship between clinical improvement and changes in connectivity in DMN might be expectable given that DMN would be related to rumination but not to core depression symptomatology as indexed in the HAM-D scale (Posner et al., 2013). Interestingly, the paper by Beall et al. (2012) found a correlation between depression symptoms improvement and rACC-rDLPFC connectivity. Given that other studies measuring connectivity between other networks also find this type of correlation (Abbott et al., 2014; Wang et al., 2014), this phenomenon should be studied in more depth.

Finally, we would like to mention that some papers hint at the existence of a causal relationship between recovery from depression symptoms and specific changes in brain connectivity. For example, the paper by Salomons et al. (2014)—where rTMS sessions were applied on dmPFC as a depression treatment—found post-treatment changes between dmPFC and other limbic structures. The paper by Abbott et al. (2014) found post-treatment changes in connectivity in the right hemisphere, which is attributed to the fact that the ECT sessions were mostly applied on the right side and unilaterally (Abbott et al., 2014). Interestingly, the paper by Liston et al. (2014) applied rMTS sessions to DLPFC but found no changes in connectivity in CCN. The paper by Heller et al. shows that treatment with duloxetine was related to changes in connectivity in DMN, but the same thing did not happen with the placebo group. Some papers suggest that some specific changes in connectivity after depression treatment might depend, partly, on the specific pharmacological treatment administered (Lisiecka et al., 2011), although the paper by Heller et al. (2013) found no differences between treatment groups in the correlation between changes in the connectivity of BA 46 and the positive affection improvement among its depressed patients. Therefore, the selection of papers suggests that part of the changes in brain connectivity experienced after depression treatment might depend—partly, at least—on the mechanisms of action of the treatment administered. This, in turn, would not only bear important clinical implications, but it would also open the door for the use of connectivity as a tool to study the mechanisms of action and therapeutic of several depression treatments.

The current paper has some limitations, too, namely the fact that it is a systematic review but not a meta-analysis. The number of studies and their heterogeneity advised us against the latter type of study. In addition, the number of papers that can be included in the review, the variety in research designs, and the variety of treatments administered among other variables render it very difficult to build a conclusive model on brain connectivity related to depression treatment. Therefore, we are unable to build an integrative model about the changes in brain connectivity after depression treatment. For these reasons, the conclusions of this paper should be considered as preliminary. Another limitation of the present work is the publication bias that usually occurs in fMRI studies (Jennings and Van Horn, 2012). In fact, disregarding reports as that from Aizenstein et al. (2009), researches in which positive association between successful antidepressant treatment and changes in brain connectivity could not be demonstrated might remain unpublished. Indeed, this and other relevant aspects could bias our review. Nevertheless, the paper also has some strength. To our knowledge, it is the only paper to review systematically treatment-related changes in brain connectivity measured through fMRI, and we can offer a few preliminary conclusions on the relationship between the treatment of depression and changes in brain connectivity. They are listed below.

The somatic treatment of depressive symptoms seems to be associated generally to changes in brain connectivity. As for DMN, in most of the studies included, it was found that this network is hyperactive in depressed patients, and that the treatment tends to normalize DMN hyperconnectivity. However, this normalization is usually incomplete, and the frontal portions of DMN might probably still present alterations of connectivity after treatment. The connectivity of cortical-limbic connections related to the emotional processing also changes after depression treatment. The studies suggest that a successful antidepressant treatment would be linked to changes in the connectivity of different sub-networks involved in negative emotional processing, and possibly also in the processing of positive emotions. Current data suggests that CCN might not undergo changes after a successful antidepressive treatment. In a rather consistent way, changes in connectivity between cortical-limbic structures correlate with an improvement of the core depressive symptoms. However, there seems to be no link between changes in the connectivity of DMN after antidepressive symptom and the improvement of the clinical symptoms of the condition. Lastly, we should point out that some papers hint at the existence of a causal relationship between recovery from depression symptoms and specific changes in brain connectivity, and also that depression treatment might depend—partly, at least—on the mechanisms of action of the treatment administered, which would have important theoretical and clinical implications.

Another strength of our work is the fact that it allows us to make a series of recommendations that, on the one hand, it might guide future researchers exploring the effect of anti-depression treatments on brain connectivity and, on the other, they would help to report some of the data of this type of study to improve its scientific quality.

1. Presenting complete sociodemographic data: As can be seen in Tables 1, 2, in various papers, these data are not sufficiently reported. However, they are essential to understand the nature of the sample on which the study was conducted.
2. Reporting the data related to the psychopathology and the psychopathological evaluation of the patients: Some of the studies comprised in the current review only mention that they had a depression, but the types of the patients' depressive disorders are not specified, nor are the criteria under which they were diagnosed. In several papers, the data on the severity of depressive symptomatology are incomplete. Sometimes even essential data are missing, such as the standard deviation of the Hamilton scale scores, before and after the treatment. In addition, many papers do not report data on the possible psychopathological comorbidities of the depressed patients. It might be advisable to present all these data in the Results Section of the articles for a better comprehension of the type of sample used in each study.
3. Increasing the types of clinical instruments used: In most of the studies reviewed, the effect of anti-depression treatment is measured through the Hamilton scale for depression and, when trying to establish correlations among changes in brain connectivity after antidepressant treatment, they are often non-significant. However, this does not necessarily mean that there is no relevant association between the clinical improvement of specific symptoms of depression and brain connectivity. For example, clinical scales such as the Beck scale, which measures symptoms of rumination among others, might show correlations between the possible changes in DMN after the treatment and improvement of clinical symptomatology. The study by Heller et al. (2013) found correlations between changes in brain connectivity and improvement of positive and negative affect. Nevertheless, despite the fact that depression is an affective disorder, affection scales are not commonly used to monitor clinical change in depressed patients. These instruments along with others—such as, for example, neuropsychological tests that measure specific cognitive domains—should be used if we mean to establish correlations between changes in connectivity and clinical improvement.
4. Conducting, as far as possible, hypothesis-driven studies: In the current review we can observe a few papers with data-driven analyses, or where the correlations of one ROI with the full brain were studied. The problem with these studies is that data interpretation is complex and, logically, the authors tend to discuss the part of the results most relevant to them, while discarding another part of the results as possible “noise.” The problem with this approach is that, as we mentioned in the introduction, and as can be seen in Tables 3, 4, the brain areas affected by depression are very diverse and, therefore, this type of study runs the risk of not interpreting or not giving relevance to changes in certain connectivity patterns after the treatment of depression that might be important.
5. Using a control group to better establish the magnitude of the change in connectivity. Pre- and post-studies allow us to see whether there are changes in brain connectivity after antidepressant treatment. However, in many of the studies, there is no control group, which prevents us from knowing the magnitude of alterations in brain connectivity and, therefore, we cannot know whether brain connectivity—or to what extent changes in brain connectivity after antidepressant treatment—shows a tendency toward the recovery of the connectivity of a healthy person. We should also bear in mind that pre- and post-treatment designs tend to overestimate the magnitude of connectivity changes after treatment. Accordingly, the lack of a control group somewhat hinders the interpretability of the data in terms of an effect size.
6. Using research designs that allow us to establish cause-effect relationships: As we mentioned above, some studies suggest the existence of a causal relationship between the treatment of depression and changes in brain connectivity. However, pre- and post-treatment designs are not the most suitable to establish this type of relationship, given the lack of adequate methodological control measures. It is suggested that, as far as possible, these studies should compare treatments among themselves, or else that short studies should be designed with sham or placebo groups. However, the latter may be difficult, as it would involve leaving a group of patients untreated, which would be disputable from an ethical point of view because these papers do not study the possible efficacy of the treatments.

We are aware that the latter two points are, to some extent, incompatible. Establishing a brain connectivity baseline and trying to establish causal relationships between treatment and changes in connectivity would involve, most of the time, designing studies with more than two groups and their corresponding follow-up. However, we do believe that, in designing future studies, they should ideally either comprise a control group, or choose a design that allows us to better research the possible causal relationships between the effect of treatment and changes in brain connectivity, like using two arms of active treatment. The current review allows us to establish that, generally, the treatment of depression is linked to changes in brain connectivity. The two most relevant questions to answer at the moment would be, on the one hand, whether antidepressant treatment is linked to the recovery of normal connectivity and to what extent, and on the other, whether there is a cause-effect relationship between the treatment of depression and the recovery of connectivity, or else if the link between both phenomena is influenced by other variables. The current state of the art does not allow us to identify bigger matters or venture more complex hypotheses on this topic. This paper clearly shows how, at the moment, a descriptive one is the best methodological approach to review the contributions of the estimations of brain connectivity models in the study of the effectiveness of treatments related to MDD. This field is no different from other similar ones, both medical and psychological. For example, the situation resembles—to a great extent—that which is perceived in disorders unrelated to the one discussed here. For instance, papers on fMRI in type 1 diabetes (Gallardo et al., 2015) show the same embryonic state as the brain connectivity effects of depression treatment, we have noted. Accordingly, the current state of affairs should be considered, in our opinion, an undeniably relevant opportunity for scientific breakthrough.

### Conflict of interest statement

The authors declare that the research was conducted in the absence of any commercial or financial relationships that could be construed as a potential conflict of interest.
